# Multi−cohort validation based on coagulation-related genes for predicting prognosis of esophageal squamous cell carcinoma

**DOI:** 10.3389/fimmu.2025.1662599

**Published:** 2025-11-26

**Authors:** Rui Wang, Weisong Zhang, Xia Li, Hao Wang, Yanan Ji, Jing Zhao, JianXiang Song, Zhongquan Yi

**Affiliations:** 1 Department of Cardiothoracic Surgery, Affiliated Hospital 6 of Nantong University, Yancheng Third People’s Hospital, Yancheng, China; 2 Department of Cardiovascular Surgery, The First Affiliated Hospital of Nanjing Medical University, Nanjing, China; 3 Department of General Medicine, Affiliated Hospital 6 of Nantong University, Yancheng Third People’s Hospital, Yancheng, China; 4 Department of Central Laboratory, Affiliated Hospital 6 of Nantong University, Yancheng Third People’s Hospital, Yancheng, China

**Keywords:** esophageal squamous cell carcinoma, coagulation-related genes, immune infiltration, prognosis signature, immunotherapy

## Abstract

**Objective:**

In malignant tumors, a hypercoagulable state is frequently observed and is intricately intertwined with cancer development and the remodeling of the immune microenvironment. Recently, the coagulation-related genes (CRGs) signature has emerged as highly significant for the prognosis and immunotherapy of patients with various cancers. Nevertheless, their application in esophageal squamous cell carcinoma (ESCC) remains uninvestigated. Here, our objective is to explore the role of the CRGs signature in forecasting prognosis and predicting patient’s response to immunotherapy.

**Methods:**

According to the prognostic CRGs, consensus clustering was utilized to stratify ESCC patients in the GSE53625 cohort into two subgroups. Subsequently, difference analysis and univariate cox analysis were conducted on the two subgroups, and a CRGs signature was constructed by leveraging these genes. Next, multiple RNA transcriptome cohorts were utilized to validate the signature. Moreover, functional enrichment, tumor mutation burden (TMB), tumor infiltration, immune function, and immunotherapy response of this signature were investigated.

**Results:**

A CRGs signature composed of six genes (PTX3, CILP, CFHR4, SULT1B1, IL5RA, and FAM151A) was constructed. This signature serves as an independent and reliable prognostic factor. Additionally, when compared with the 32 prognostic signatures previously reported, the CRGs signature exhibited superior performance in the ESCC prognostic cohorts. Additionally, we found that high-risk ESCC exhibited higher immune infiltration, lower TMB, higher TIDE, and a lower proportion of immunotherapy response. *In vitro* experiments have shown that the gene SULT1B1, which exhibits the highest accuracy in predicting tumor status, significantly inhibited the proliferation and metastasis.

**Conclusions:**

We constructed and validated a robust CRGs signature. Moreover, as one of the model CRGs, the tumor-suppressive role of SULT1B1 in ESCC was experimentally verified *in vitro*. These results provide novel insights into enhancing the prognosis of ESCC and formulating treatment strategies.

## 1 Introduction

With a high incidence worldwide, esophageal cancer (EC) is a major cause of deaths associated with cancer (1–3). Esophageal squamous cell carcinoma (ESCC) constitutes the prevailing type, comprising nearly 90% of cases (4). Despite the progress in treatments, the accessible treatment options for advanced ESCC remain limited, and the cure rates are comparatively low (3, 5, 6). Recently, with the deepening comprehension of tumor immune microenvironment (TME), immunotherapy has witnessed rapid development (7). Nevertheless, due to the significant heterogeneity in ESCC, merely a small proportion of cases exhibited a favorable response to immunotherapy (8). The TME exists within a complex and dynamic multicellular environment (9, 10). Conducting a thorough and detailed investigation of the TME in ESCC patients is instrumental to elucidating the immune landscape of ESCC. This has important practical significance for evaluating patients’ responsiveness to immunotherapy and formulating new strategies in immunotherapy (11).

The coagulation system, a sophisticated biological process, guarantees efficient hemostasis, maintains blood flow, and simultaneously prevents excessive bleeding (12–15). In malignant tumors, a hypercoagulable state is often observed (16, 17). This state may give rise to venous thromboembolism, thereby inducing local hypoxia and necrosis. Subsequently, it further triggers the proliferation of microvascular, the migration of tumor cells, and the remodeling of the TME (18–21). Recently, the significance of coagulation-related genes (CRGs) in prognostic prediction and predicting the response to immunotherapy has drawn attention (15, 16, 21–27). For instance, Wu et al. (15) constructed a CRGs signature for colon adenocarcinoma that reliably predicted both prognosis and treatment outcomes. He et al. (27) formulated a CRGs signature in hepatocellular carcinoma and analyzed its role in prognosis, immunotherapy, and chemotherapy response. Nevertheless, its role in ESCC remains unknown. Hence, it is essential to further explore the effects of the CRGs signature on ESCC, especially the prognosis prediction potential and its influence on clinical treatment decisions.

In this study, six transcriptome cohorts from TCGA and GEO databases were obtained for analysis. Subsequently, machine learning algorithms were employed to construct a CRGs prognostic signature, and the role of potential intervention targets in ESCC was verified through *in vitro* experiments. Our findings contributed to the advancement of prognostic biomarkers, offered a novel perspective on the involvement of CRGs in ESCC, and furnished new information for precision treatment.

## 2 Materials and methods

### 2.1 Samples and data collection

**Figure 1:**
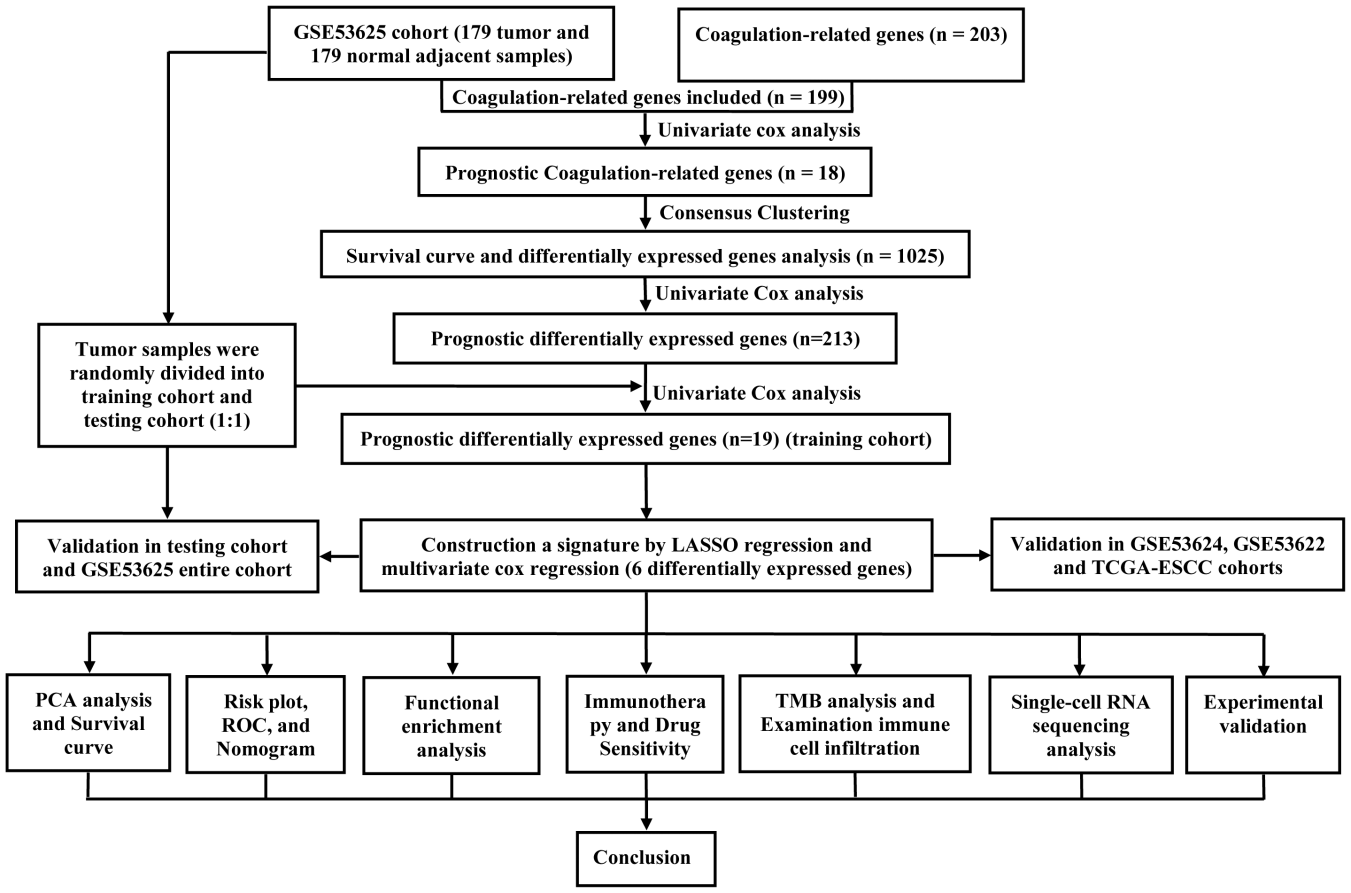
The flow chart of research design. ESCC, esophageal squamous cell carcinoma; PCA, principal component analysis; ROC, receiver operating characteristic; TMB, tumor mutational burden.

**Figure 1** exemplified the investigation process. In this study, four transcriptome cohorts encompassing complete overall survival rate (OS) information and clinical information (TCGA-ESCC, GSE53625, GSE53624, and GSE53622) were obtained from TCGA and GEO databases. Besides, two other transcriptome cohorts from the GEO database were included (GSE20347and GSE38129). All the six transcriptome cohorts undergo a log-2 transformed to ensure normalization. Subsequently, to eliminate the batch effect, the ComBat algorithm was employed, and the extent of correction was examined via principal component analysis (PCA, **Supplementary Figures 1A, B**).

The pre-preprocessing of ESCC single-cell data (with a sample size of n = 7 for ESCC)) from the GSE145370 dataset was conducted using the Seurat v4 and Harmony (version 1.0). Low-quality cells with a mitochondrial gene proportion >20% and a gene count of <200 and >6000 were removed. To visualize the cell clusters, the t-distributed random neighbor embedding (t-SNE) was employed.

Furthermore, a curated list of 203 CRGs (**Supplementary Table 1**), such as SERPINE1, F3, and THBD, were obtained from the Gene Set Enrichment Analysis (GSEA) database. These genes originated from the hsa04610 pathway (complement and coagulation cascade) and the hsa04611 pathway (platelet activation). Both of these pathways are closely linked to coagulation and relevant processes.

### 2.2 Construction and validation of the CRGs signature

Initially, a univariate cox analysis of 203 CRGs in the GSE53625 cohort was conducted to identify prognostic CRGs. Next, the ‘ConsensusCluster Plus’ R package was utilized for consensus clustering with these prognostic CRGs. The optimal cluster count was determined by making use of the CDF curve, consensus score matrix, and PAC score. To detect prognostic DEGs between clusters, the ‘limma’ package (with a |logFC| > 0.585 and a *p*-value < 0.05) and univariate cox regression were utilized (28). In this research, the samples from the GSE53625 cohort were randomly assigned to a training cohort with 90 samples and a testing cohort encompassing 89 samples through the utilization of the R package ‘caret’. The clinical information of the two cohorts was shown in **Table 1**. Here, the CRGs signature was formulated with GSE53625 training cohort and verified in diverse cohorts, including the GSE53625 testing, the entire GSE53625, the GSE53624, the GSE53622, and the TCGA-ESCC cohorts. In the GSE53625 training cohort, hub prognostic DEGs were screened out through univariate cox regression, LASSO regression, and multiple cox regression analysis with a stepwise approach. Each sample’s risk score was evaluated utilizing the formula: Risk score =∑i=EXP (i) ×Coef (i). Next, the median score of each cohort was tallied, and each ESCC sample was categorized as high- or low-risk based on the median score. We utilized the ‘Survival’ R package and employed the ‘survival ROC’ package to evaluate the predictive capability (29). Additionally, cox regression and nomogram analysis were performed in conjunction with the clinical features.

**Table 1:** Comparisons of patient characteristics between training and testing cohorts.

Characteristics	Total (*n* = 179)	Training set (*n* = 90)	Testing set (*n* = 89)	*P-*value
Age				
≤ 60	99 (55.31%)	48 (53.33%)	51 (57.30%)	0.593
> 60	80 (44.69%)	42 (46.67%)	38 (42.70%)	
Gender				
Male	146 (81.56%)	72 (80.00%)	74 (83.15%)	0.587
Female	33 (18.44%)	18 (20.00%)	15 (16.85%)	
T stage				
T1	12 (6.71%)	6 (6.67%)	6 (6.74%)	0.518
T2	27 (15.08%)	10 (11.11%)	17 (19.10%)	
T3	110 (61.45%)	58 (64.44%)	52 (58.43%)	
T4	30 (16.76%)	16 (17.78%)	14 (15.73%)	
N stage				
N0	83 (46.37%)	43 (47.78%)	40 (44.94%)	0.877
N1	62 (34.64%)	30 (33.33%)	32 (35.96%)	
N2	22 (12.29%)	10 (11.11%)	12 (13.48%)	
N3	12 (6.70%)	7 (7.78%)	5 (5.62%)	
TNM stage				
I	10 (5.59%)	7 (7.78%)	3 (3.37%)	0.438
II	77 (43.02%)	38 (42.22%)	39 (43.82%)	
III	92 (51.39%)	45 (50.00%)	47 (52.81%)	

### 2.3 Functional enrichment analysis

Researchers executed relevant enrichment analysis on DEGs utilizing ‘clusterProfiler’ R package (30), encompassing GO and KEGG analyses. Moreover, to analyze potential modifications in signaling pathways, Hallmark gene sets were applied in GSEA.

### 2.4 Prediction of immunotherapy response and analysis of gene mutation data and drug sensitivity

To analyze the differences between risk groups, the immune, estimate, stromal, and tumor purity scores were calculated by utilizing the ESTIMATE method. Moreover, ssGSEA algorithms were utilized to assess both the overall immune function and the immune cell infiltration.

Furthermore, the software package “maftools” was utilized to obtain tumor mutation burden (TMB) data (31). Meanwhile, the microsatellite instability (MSI) score was retrieved from the public data of TCGA (**Supplementary Table 2**). Besides, each patient’s TIDE scores were from the online website (32). Moreover, “oncoPredict” package was utilized to perform drug sensitivity analysis (*p* < 0.05) (33).

### 2.5 Cell lines

Three ESCC cell lines, KYSE30, KYSE150, and KYSE410, were sourced from Pricella (Wuhan, China), and cultured in RPMI-1640 (Pricella) supplemented with 10% FBS (Pricella) and 1% penicillin-streptomycin (Pricella). These cells were cultured at 37 °C in 5% CO2. The siRNAs for SULT1B1 (si- SULT1B1-1/-2/-3/-4, details in **Supplementary Table 3**), negative control (si-NC), and SULT1B1 overexpression plasmid were provided by GenePharma (Shanghai, China). And to transfect the cells, Lipofectamine 3000 regent (L3000015, Invitrogen, USA) was employed. Forty-eight hours post-transfection, cells were harvested to analyze the proteins levels, apoptosis, and cell cycle. Annexin V-FITC Apoptosis Detection Kit (Beyotime, C1062, China) and Cell cycle Analysis Kit (Beyotime, C1052, China) were used for apoptosis and cell cycle assay.

### 2.6 Western blotting

To prepare protein extracts, cells were lysed with a mixture of RIPA buffer (Beyotime, P0013B, China) and 1% PMSF (Beyotime, ST505, China). The protein samples were then denatured, and the proteins were resolved by SDS-PAGE gel. Subsequently, the proteins were transferred onto a PVDF membrane, which was blocked with 5% skim milk for 2 hours. Next, the membrane was incubated with primary antibodies, namely SULT1B1 (1:500; Proteintech, 16050-1-AP, China), E-cadherin (1:5000; Proteintech, 20874-1-AP, China), Vimentin (1:1000; Beyotime, AF1975, China), and GAPDH (1:1000; Servicebio, GB15004-100, China). After incubation with the secondary antibody, the bands were developed by means of the ECL developer (Beyotime, E422-01, China).

### 2.7 CCK-8 assay

A density of 2000 cells per well was utilized for seeding in 96-well plates. After that, to each well, 10 µL of CCK-8 solution in a serum-free medium was added and incubated at 37 °C for one hour. Next, measurements of absorbance were conducted at a wavelength of 450 nm, with the optical density (OD) being recorded at the same daily interval.

### 2.8 Cell migration assay

A volume of 100 µL of serum-free medium, which contained 5 × 10^4^ cells, was seeded on the top chamber of the transwell. Concurrently, 600 µL of complete medium was inoculated into the bottom chamber. Following 24 hours of incubation, the cells were stained with a 0.5% crystal violet solution and then counted under a microscope.

### 2.9 Scratch wound healing assay

Utilize the sterile plastic pipette to scrape the transfected cells. Subsequently, wash the cells twice with PBS, and then replenish the culture with fresh medium. Images of the scratch were captured under a microscope at 0 and 24 hours post-treatment.

### 2.10 Statistical analysis

For statistical analysis and graphing, R 4.3.1 and GraphPad Prism 8 were utilized. Differences between two groups were analyzed utilizing Student’s t-test or Wilcoxon’s rank sum test. For statistical analysis above two groups, a one-way ANOVA test was performed. A *p* < 0.05 was considered statistically significant.

## 3 Results

### 3.1 Identification and establishment of CRGs signature

**Figure 2:**
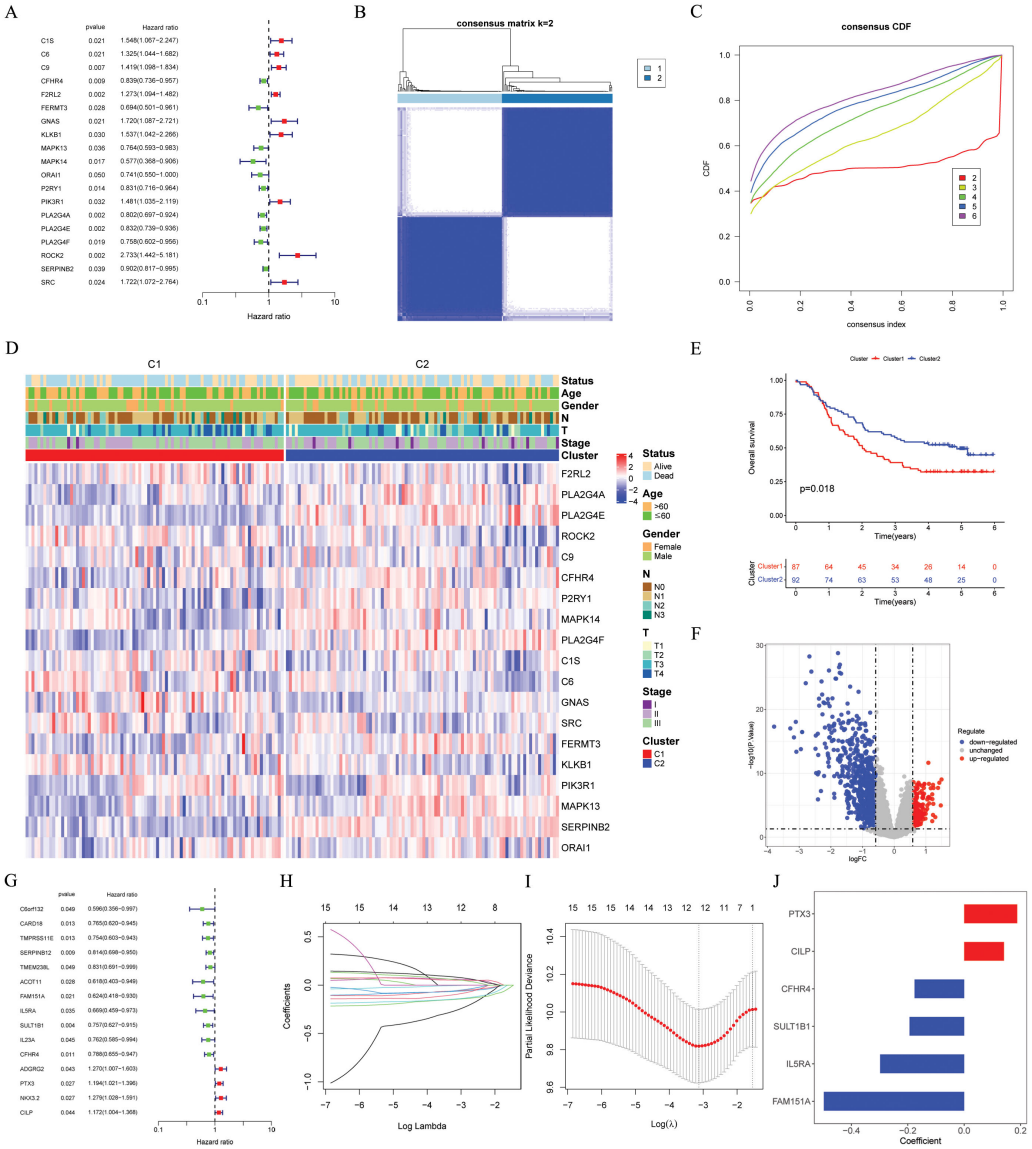
Identification and construction of the CRGs signature. **(A)** 19 prognostic CRGs were identified through univariate cox analysis (*p* < 0.05). **(B)** The consensus score matrix of the GSE53625 cohort when k = 2. **(C)** The CDF curves of consensus matrix for each k, where k ranges from 2 to 6. **(D)** A heatmap depicted the expression levels of 19 prognostic CRGs, accompanied by clinical characteristic annotations for each cluster. **(E)** The Kaplan-Meier survival curve depicted significant different overall survival between the two clusters (*p* = 0.018). **(F)** A volcano plot depicted DEGs between the two clusters with criteria of |logFC| > 0.585 and *p* value < 0.05. **(G)** Univariate cox regression analysis was conducted to identify prognostic DEGs with a significance level of *p* < 0.05. **(H, I)** The coefficient profile of prognostic DEGs was determined by Lasso regression analysis. The optimal λ was achieved when the partial likelihood deviance reached the minimum value. **(J)** The coefficients of the 6 prognostic DEGs (PTX3, CILP, CFHR4, SULT1B1, IL5RA and FAM151A), which were utilized to construct the CRGs signature, were obtained from multivariate cox analysis. CRGs, coagulation-related genes; DEGs, different expression genes.

Univariate cox regression was performed on 203 CRGs, and 19 prognostic CRGs were filtered out (**Figure 2A**). We then proceeded with a consensus cluster analysis, and identified the optimal number k was 2 (**Figures 2B, C**). The differences between the two consensus clusters (C1 and C2) in terms of 19 prognostic CRGs and clinical characteristics were illustrated in **Figure 2D**. According to the KM analysis, there were marked prognostic differences between the two clusters (**Figure 2E**). Next, 1025 DEGs (**Figure 2F**; **Supplementary Table 4**) and 215 prognostic DEGs were filtered out. No notable variations were observed between the two cohorts. **Figure 2G** illustrated the 36 prognostic DEGs in the training cohort. Subsequently, six model DEGs were screened out through the utilization of LASSO regression (**Figures 2H, I**) and multivariate Cox regression. **Supplementary Table 5** presented the univariate and multivariate results of these six model DEGs. Thereafter, a CRGs signature was established (**Figure 2J**) according to the formula: Risk score = PTX3* 0.18815 + CILP * 0.14112 + CFHR4* (-0.17575) + SULT1B1* (-0.19359) + IL5RA * (-0.29789) + FAM151A * (-0.49759).

### 3.2 Evaluation of the CRGs signature

**Figure 3:**
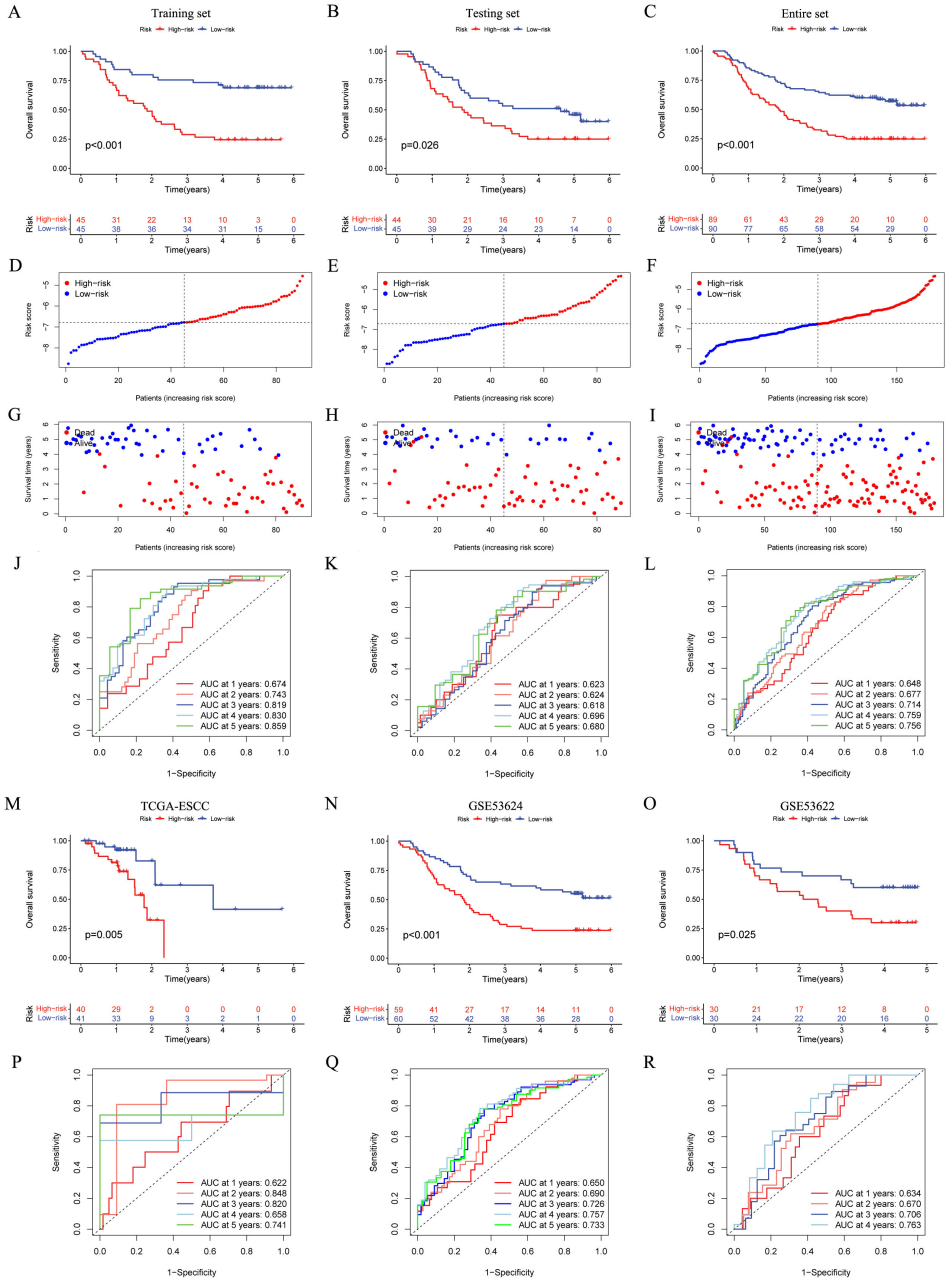
Establishment and validation of the CRGs signature in both internal and external cohorts. **(A–C)** Overall survival of patients in different risk groups in the GSE53625 training (n = 90, *p* < 0.001), testing (n = 89, *p* = 0.026), and entire (n = 179, *p* < 0.001) cohorts was analyzed, with low CRGs group showing better outcomes. **(D–I)** The distribution of risk scores **(D–F)** and OS status **(G–I)** for each patients in the GSE53625 training, testing, and entire cohorts. **(J–L)** Time-dependent ROC curves for predicting 1-, 2-, 3-, 4-, and 5-year OS in the GSE53625 training, testing, and entire cohorts. **(M–R)** Kaplan–Meier analysis and time-dependent ROC curves in three external validation cohorts: TCGA-ESCC, GSE53624, and GSE53622. CRGs, coagulation-related genes; OS, overall survival; ROC, Receiver operating characteristic; ESCC, esophageal squamous cell carcinoma.

The OS status and risk score distribution for GSE53625 training, testing, and entire cohorts were depicted in **Figures 3D–I**. Besides, within the aforementioned three cohorts, notably poorer prognosis was detected in high-risk group (**Figures 3A–C**). Additionally, the validity of signature prediction was corroborated by the TCGA-ESCC, GSE53624, and GSE53622 cohorts (**Figures 3M–O**). The time-dependent ROC of the signature was plotted (**Figures 3J–L, P–R**). The results indicated that across all cohorts, the AUC values at 1–5 years all exceeded 0.6. This observation evidenced high specificity and sensitivity. Furthermore, a random-effects meta-analysis was conducted on the hazard ratios (HR) across the above four cohorts (GSE53625, TCGA-ESCC, GSE53624, and GSE53622), and the results showed that the CRGs signature was a risk factor for OS in ESCC (HR = 1.73, 95% CI = 1.52 - 1.97, *I^2^* = 0, illustrated in **Supplementary Figure 1C**).

**Figure 4:**
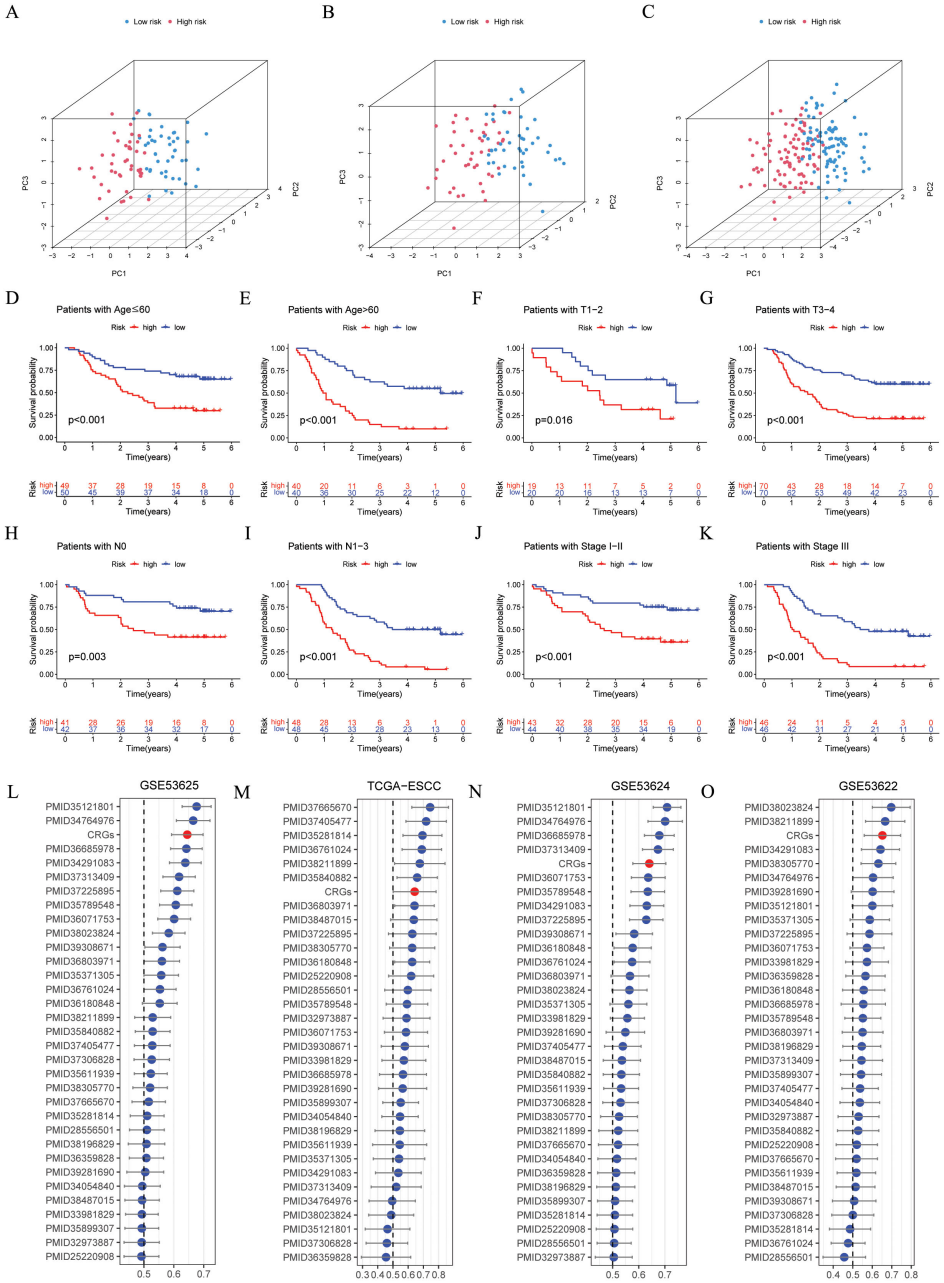
Evaluation of the CRGs signature performance. **(A–C)** PCA analyses for the CRGs signature were conducted in the GSE53625 training (n = 90), testing (n = 89), and entire (n = 179) cohorts. **(D–K)** Kaplan–Meier curves of OS according to the CRGs score in the GSE53625 subgroup **(D)** patients with Age ≤ 60 years, *p* < 0.001; **(E)** patients with Age > 60 years, *p* < 0.001; **(F)** patients with T1-2, *p* = 0.016; **(G)** patients with T3-4, *p* = 0.001; **(H)** patients with N0, *p* = 0.003; **(I)** patients with N1-3, *p* < 0.001; **(J)** patients with Stage I- II, *p* < 0.001; **(K)** patients with Stage III, *p* < 0.001, with low CRGs group showing better outcomes. **(L–O)** C-index analysis CRGs and 32 previously published signatures in GSE53625 (n = 179), TCGA-ESCC (n = 81), GSE53624 (n = 119), and GSE53622 (n = 60) cohorts. CRGs, coagulation-related genes; PCA, principal component analysis; OS, overall survival; ESCC, esophageal squamous cell carcinoma.

**Figures 4A–C** illustrated that the CRGs signature shows a clear grouping effect as revealed by PCA. Moreover, we investigated the influence of clinical features on the CRGs signature, as depicted in **Figures 4D–K**, within subgroups including age, T, N and stage, the prognosis of the low-risk group was better. Furthermore, we conducted a comparison of the CRGs signature with 32 previously published prognostic signatures. The findings indicated that in the GSE53625, TCGA-ESCC, GSE53624, and GSE53622 cohorts, the CRGs signature was more effective in comparison to other published signatures (**Figures 4L–O**). In conclusion, these above results emphasized the remarkable predictive accuracy of CRGs signature in forecasting the prognosis of ESCC patients.

### 3.3 Independent prognosis analysis of CRGs signature and establishment of the Nomogram model

**Figure 5:**
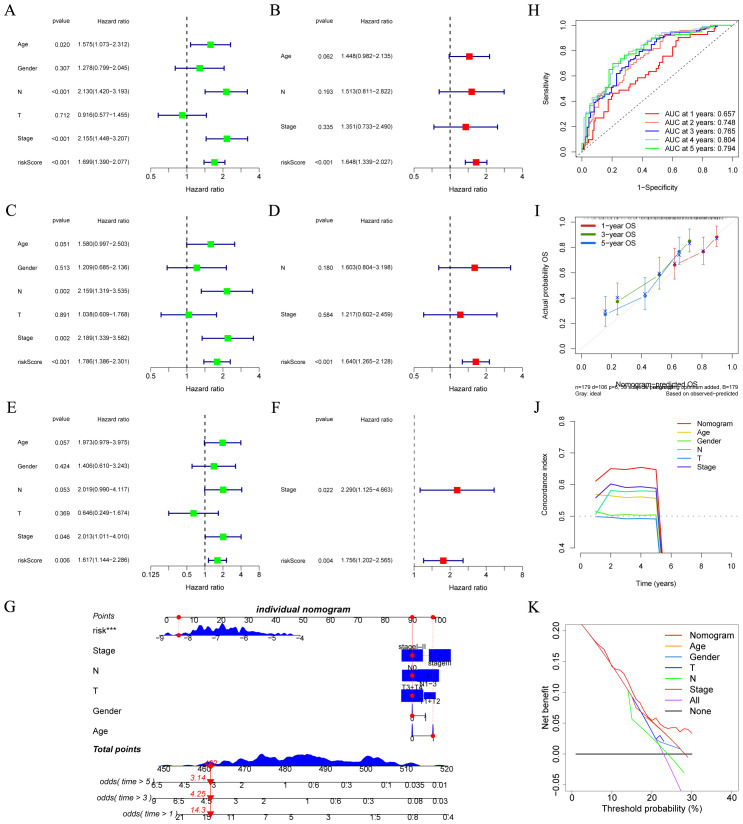
Independent prognostic analysis and construction of a nomogram. **(A–F)** Based on univariate and multivariate cox analysis, CRGs signature was an independent prognostic risk factor in the GSE53625 (**A, B**, n = 179), GSE53624 (**C, D**, n = 119), and GSE53622 (**E, F**, n = 60) cohorts. **(G)** A nomogram was established based on the CRGs signature in the GSE53625 cohort. **(H)** ROC curves presenting the prediction performance of the nomogram in 1-, 2-, 3-, 4-, and 5-year OS. **(I)** The calibration curve of the nomogram for OS at 1, 3, and 5 year **(J)** A comparison of the C index was made between the nomogram and other clinical features. **(K)** Decision curve analysis presented the net benefit by applying the nomogram and other clinical features. CRGs, coagulation-related genes; OS, overall survival; ROC, Receiver operating characteristic. ***p < 0.001.

We utilized univariate and multivariate cox analyses in the GSE53625 (**Figures 5A, B**), GSE53624 (**Figures 5C, D**), and GSE53622 (**Figures 5E, F**) cohorts to explore the prognostic implications of CRGs signature alongside various clinical features. In the cohorts mentioned above, the CRGs signature was demonstrated to be an independent prognostic risk factor (*p* < 0.05), emphasizing its significant prognostic potential. Next, a nomogram was developed that integrates risk scores with several clinical features, illustrated in **Figure 5G**. Time-dependent ROC analysis indicated that this nomogram possessed high sensitivity and specificity (**Figure 5H**). The calibration curve verified the feasibility of this nomogram in practical settings (**Figure 5I**). The outcomes of DCA indicated and the C index indicated that, in comparison to other clinical features, this nomogram possessed stronger predictive power and a higher net clinical benefit (**Figures 5J, K**). Overall, this nomogram holds the potential to emerge as an effective instrument for the accurate prognosis of patients with ESCC.

### 3.4 Function enrichment analysis

**Figure 6:**
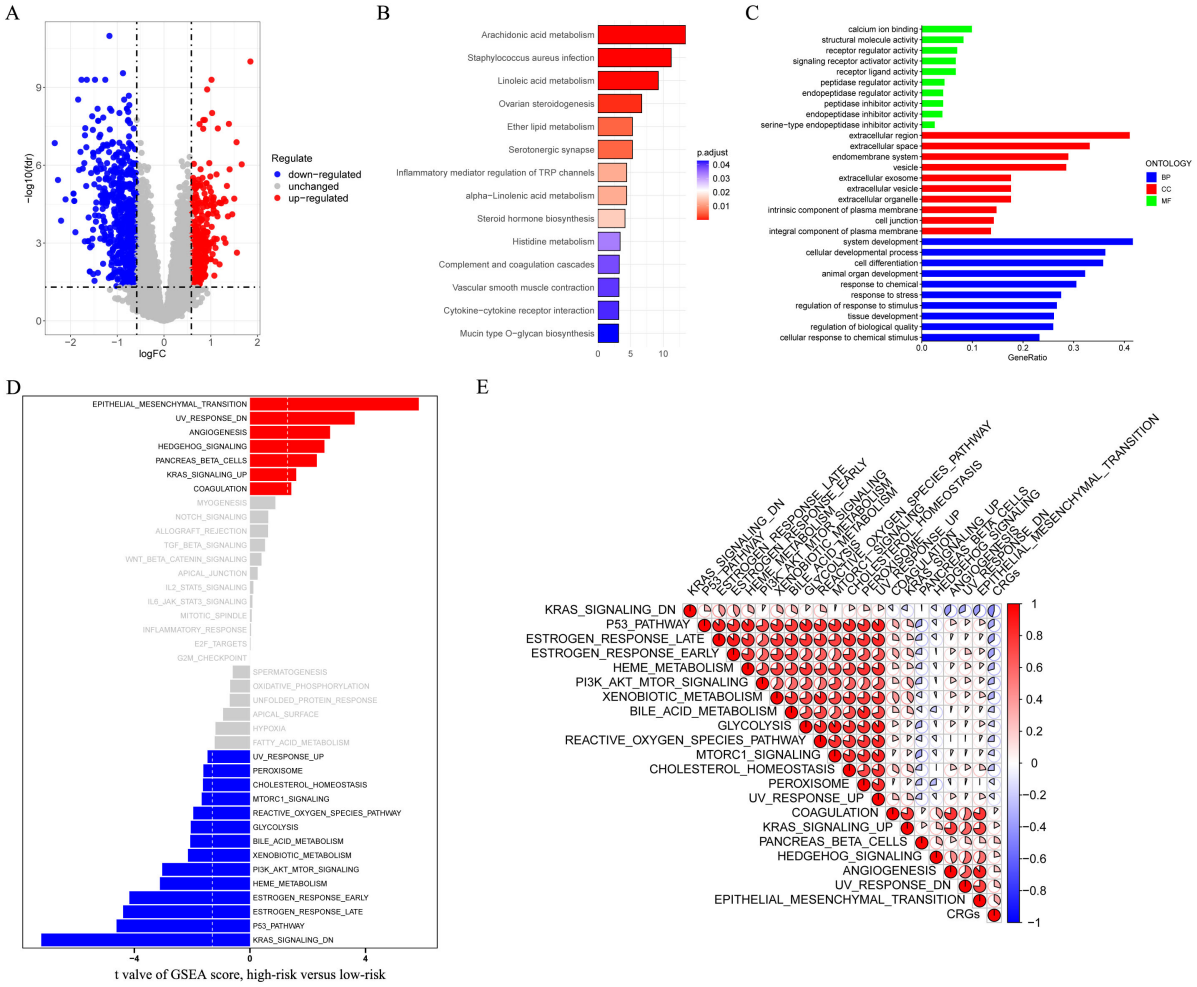
Functional enrichment analyses. **(A)** The volcano plot showing the DEGs between different risk groups in the GSE53625 cohort (n = 179) with criteria of |logFC| > 0.585 and FDR < 0.05. **(B, C)** KEGG and GO enrichment analyses revealing the potential pathways enriched by the DEGs between different risk groups. **(D)** The differences in hallmark pathway activities scored by GSEA between different risk groups. **(E)** The correlation between the risk score and hallmark pathway activities scored by GSEA. DEGs, different expression genes. KEGG, Kyoto Encyclopedia of Genes and Genomes; GO, Gene Ontology; GSEA, Gene Set Enrichment Analysis.

The DEGs (illustrated in **Figure 6A** and **Supplementary Table 6**) were mainly concentrated in “Inflammatory mediator regulation of TRP channels” and “Complement and coagulation cascades” (as shown in **Figure 6B**). GO analysis reveals a notable enrichment within the domain of biological process (BP), specifically in relation to “cellular developmental process” and “cell differentiation” (**Figure 6C**). Additionally, the GSEA analysis revealed that the high-risk groups predominantly display the activation of multiple cancer-associated signaling pathways, such as “UV RESPONSE DN” and “EPITHELIAL MESENCHYMAL TRANSITION”. Alternatively, the low-risk group was predominantly marked by “KRAS SIGNALING DN” and “P53 PATHWAY” (**Figure 6D**). The correlation analysis between CRGs scores and hallmarks pathway scores further indicated that CRGs scores was strongly associated with cancer-related biological processes and metabolic pathways (**Figure 6E**). To sum up, these results suggested that the activation or inhibition of these pathways might give rise to distinct prognostic outcomes observed in different CRGs signature subgroups.

### 3.5 Analysis of tumor microenvironment

**Figure 7:**
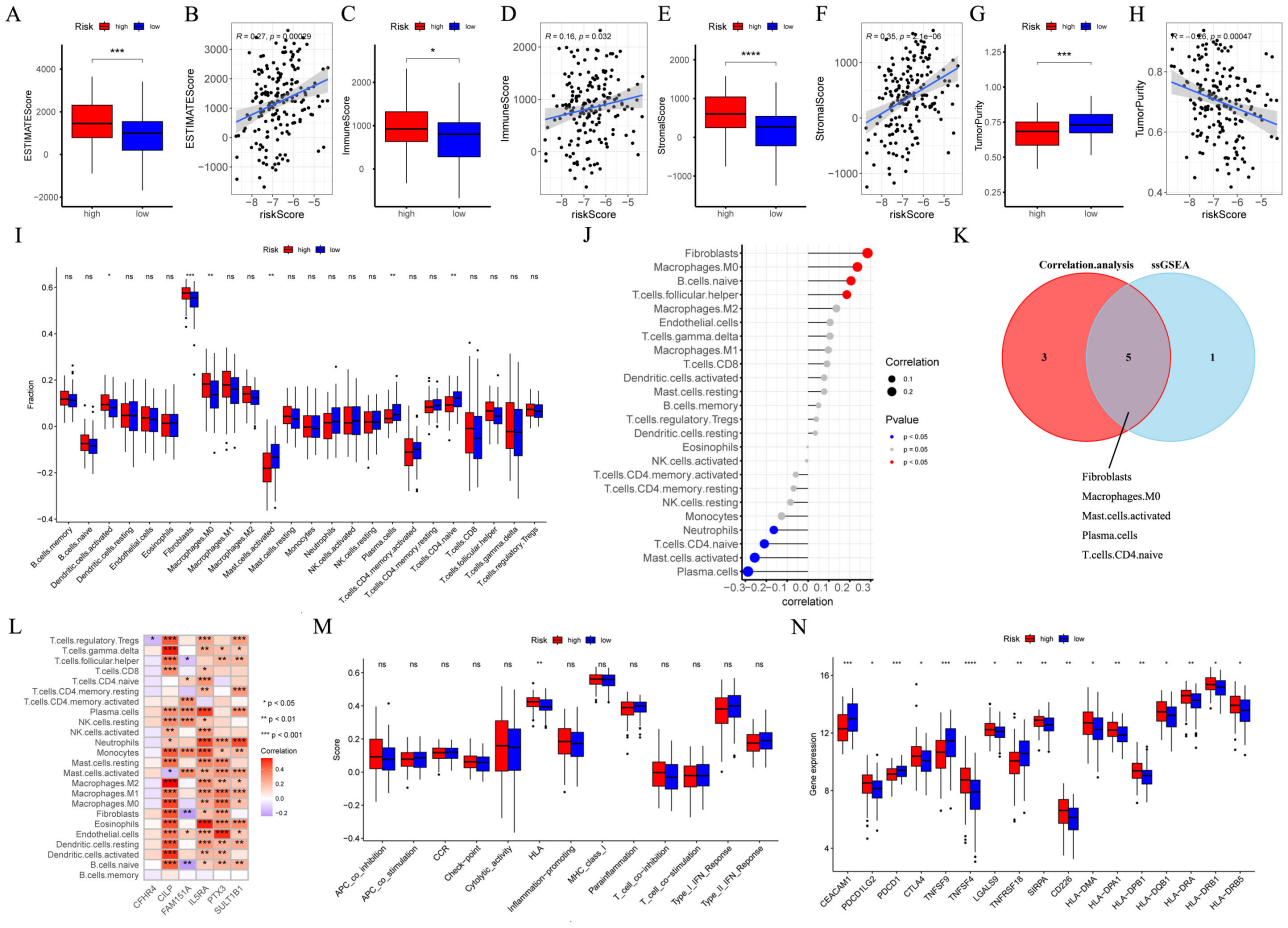
The immune landscape associated with the CRGs signature in ESCC. In the GSE53625 cohort (n = 179), **(A–H)** the Wilcoxon’s rank sum test and correlation analysis were employed to quantitatively assess the distinct immune statuses between risk groups in terms of the immune score, stromal score, estimate score, and tumor purity. **(I)** The ssGSEA algorithm was employed to analyze the differences in immune cells between different risk groups. **(J)** Pearson correlation analysis was conducted to evaluate the correlations between immune cells and risk scores. **(K)** A Venn plot depicted the intersection of the ssGSEA algorithm and correlation analysis. **(L)** Pearson correlation analysis was conducted to evaluate the correlations between immune cells and 6 model CRGs. **(M)** The ssGSEA algorithm was used to analyze differences in immune functions between different risk groups. **(N)** Box plot of expression difference of 17 immune checkpoints between different risk groups. CRGs, coagulation-related genes; ESCC, esophageal squamous cell carcinoma; ssGSEA, single sample gene set enrichment analysis. *: *p* < 0.05, **: *p* < 0.01, ***: *p* < 0.001, ****: *p* < 0.0001.

Patients with high-risk ESCC exhibited notably elevated immune, stromal, and estimate scores, in conjunction with decreased tumor purity score (**Figures 7A, C, E, G**). Besides, the risk score was positively correlated with immune, estimate, and stromal scores (**Figures 7B, D, F**). In contrast, a negative correlation was observed in tumor purity (**Figure 7H**). By utilizing the ssGSEA algorithm and the Wilcoxon test, notable differences were found (**Figure 7I**). Specifically, activated dendritic cells, fibroblasts, and macrophages M0 were notably more common in patients with high-risk ESCC. In contrast, in low-risk ESCC patients, there was a higher abundance of activated mast cells, naive CD4 T cells, and plasma cells. Besides, Pearson correlation analysis pinpointed 8 immune cells (*p* < 0.05, **Figure 7J**). Moreover, five intersecting immune cell types were identified (**Figure 7K**). Then, researchers evaluated the associations among the 6 CRGs and immune cells (**Figure 7L**). Thereafter, the two risk groups exhibited disparities in HLA enrichment (**Figure 7M**). Finally, an analysis of immune checkpoints (ICs) was performed, and seventeen ICs exhibited notable (*p* < 0.05, **Figure 7N**). These findings underscored the distinctions in immune cell infiltration between CRGs signature subgroups.

### 3.6 Comparison of TMB and Immunotherapy response in high- and low-risk groups

**Figure 8:**
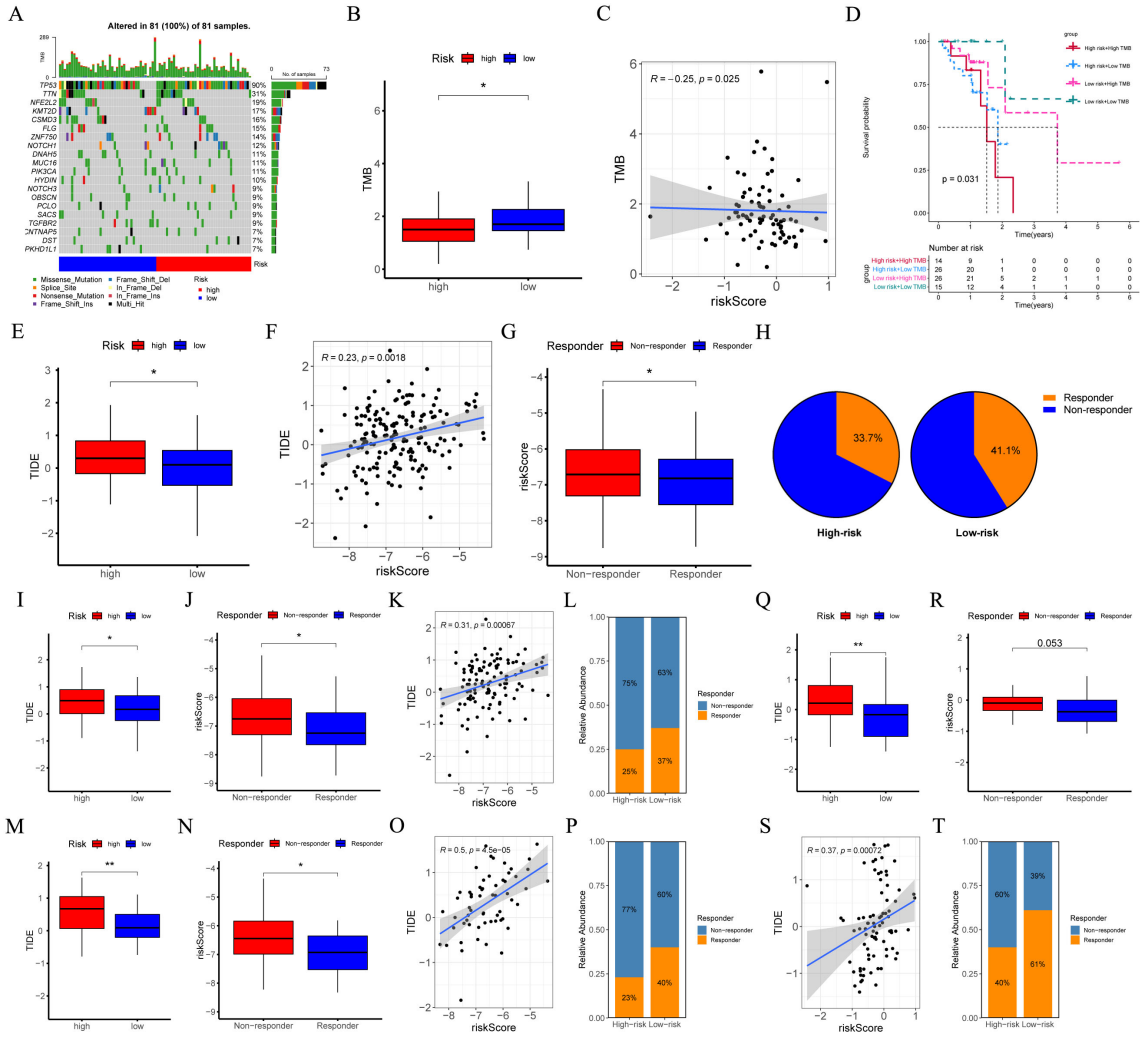
Evaluation of TMB and responsiveness to immunotherapy between different risk groups. **(A)** The waterfall plot of the somatic mutation landscape within high- and low-risk patients in the TCGA-ESCC cohort (n = 81). **(B)** Boxplots depicted the difference in TMB between high- and low-risk groups. **(C)** The correlation scatter plot depicted the relationship between TMB and risk score. **(D)** The Kaplan-Meier survival curve depicted different overall survival (*p* = 0.031) across four subgroups (high-risk and high-TMB, high-risk and low-TMB, low-risk and high-TMB, low-risk and low-TMB). **(E, I, M, Q)** Boxplots of the difference in TIDE between the high- and low-risk groups across GSE53625 (n = 119), GSE53624 (n = 119), GSE53622 (n = 60), and TCGA-ESCC (n = 81) cohorts. **(F, K, O, S)** The scatter plot showed the correlation between risk score and TIDE across GSE53625, GSE53624, GSE53622, and TCGA-ESCC cohorts. **(G, J, N, R)** Boxplots of the difference in risk score between non-response and response groups across GSE53625, GSE53624, GSE53622, and TCGA-ESCC cohorts. **(H, L, P, T)** The percentages of immunotherapy responders in the high-risk group compared to the low-risk group across GSE53625, GSE53624, GSE53622, and TCGA-ESCC cohorts. TMB, tumor mutational burden; ESCC, esophageal squamous cell carcinoma; TIDE, tumor immune dysfunction and exclusion. *: *p* < 0.05, **: *p* < 0.01.

Initially, in the TCGA-ESCC cohort, the waterfall plot was employed to depict the somatic mutation landscape (**Figure 8A**). Subsequent analysis unveiled patients within low-risk subgroup possessed higher TMB levels (**Figure 8B**), and the risk score was inversely related to TMB (R = -0.25, *p* = 0.025, **Figure 8C**). Moreover, the results suggested that patients in the “high risk + high TMB” category exhibited the poorest prognosis (**Figure 8D, p** = 0.031). Furthermore, we assessed the MSI score between different risk groups. No significant disparity in MSI score between different risk groups was detected (**Supplementary Figure 1D**), and no significant correlation existed between the risk score and the MSI score (**Supplementary Figure 1E**).

We employed TIDE to assess the potential of the immunotherapy response between different risk groups. Analyses of the GSE53625, GSE53624, GSE53622, and TCGA-ESCC cohorts revealed that, when compared with low-risk ESCC patients, the TIDE score in high-risk ESCC patients was relatively higher. (**Figures 8E, I, M, Q**). Moreover, as opposed to the responder group, the average risk score of the non-responder group exhibited an upward tendency (**Figures 8G, J, N, R**). Additionally, a positive correlation was identified (**Figures 8F, K, O, S**). Furthermore, in the aforementioned four cohorts, a higher proportion of patients in the low-risk group were predicted to respond effectively to immunotherapy (**Figures 8H, L, P, T**). These findings indicated that the CRGs signature hold substantial potential in predicting the response to immunotherapy.

### 3.7 Drug sensitivity analysis

**Figure 9:**
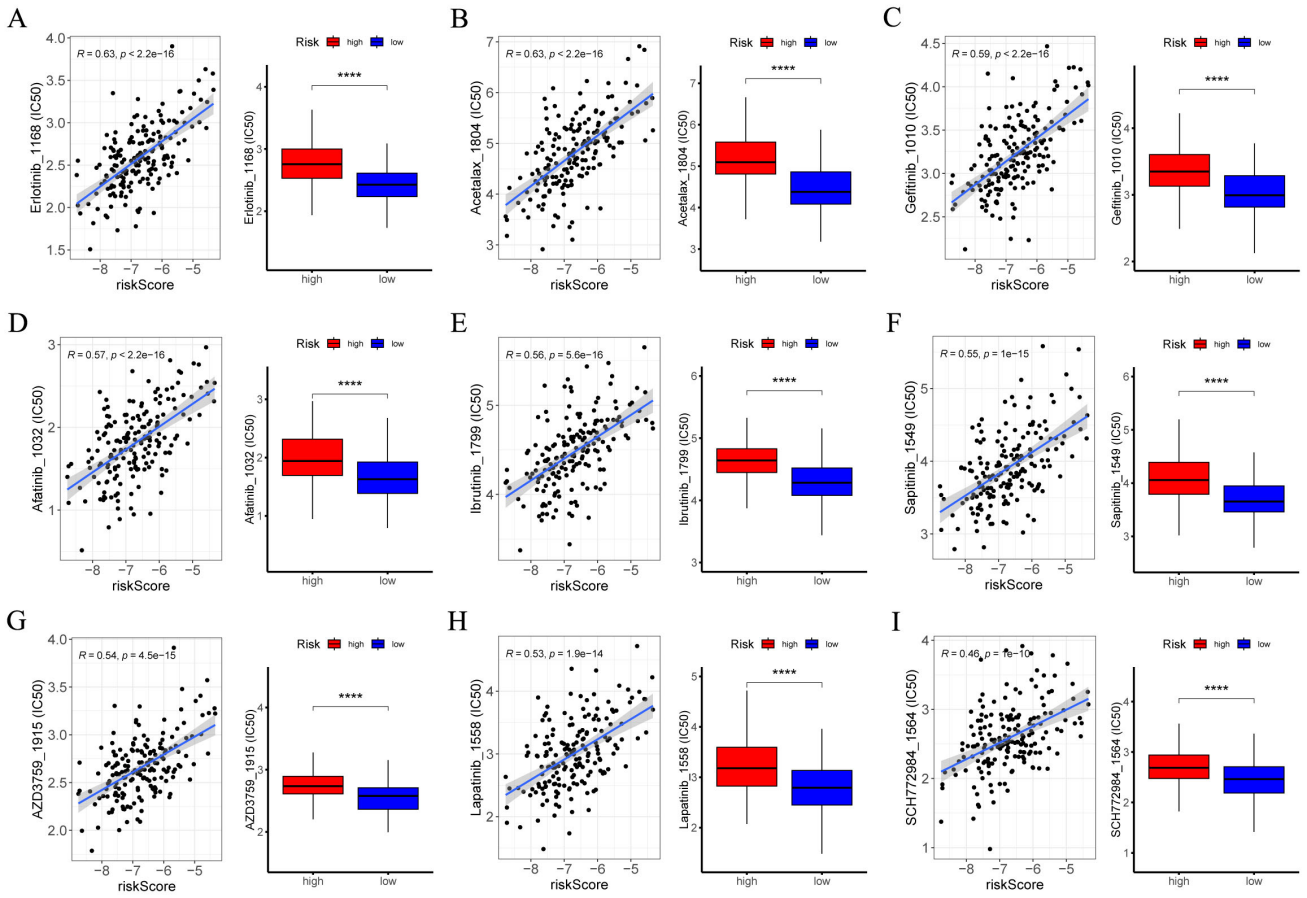
Exploration of drug compounds targeting the CRGs. In the GSE53625 cohort (n = 179), **(A–I)** correlation scatter plot depicting the relationship of IC50 of the top 9 candidate drugs and risk score, and boxplots depicting the difference in IC50 of candidate drugs between high- and low-risk groups, with statistical significance assessed via the Wilcoxon rank sum test. Pearson correlation analysis was performed to assess the correlations between risk score and candidate drugs. CRGs, coagulation-related genes; IC50, the half-maximal inhibitory concentration. ****: *p* < 0.0001.

Through Pearson correlation analysis, nine drugs with the strongest correlation between IC50 values and risk scores were screened out. Patients with high-risk ESCC showed higher sensitivity to Erlotinib, Acetalax, Gefitinib, Afatinib, Ibrutinib, Sapitinib, AZD3759, Lapatinib, and SCH772984 (**Figures 9A–I**). The results of the Wilcoxon test revealed that notable statistical differences were identified (*p* < 0.05). The findings may provide insights into the selection of potential treatment options to inhibit the malignant progression of cancer.

### 3.8 Identification of model CRGs in single−cell transcriptome

**Figure 10:**
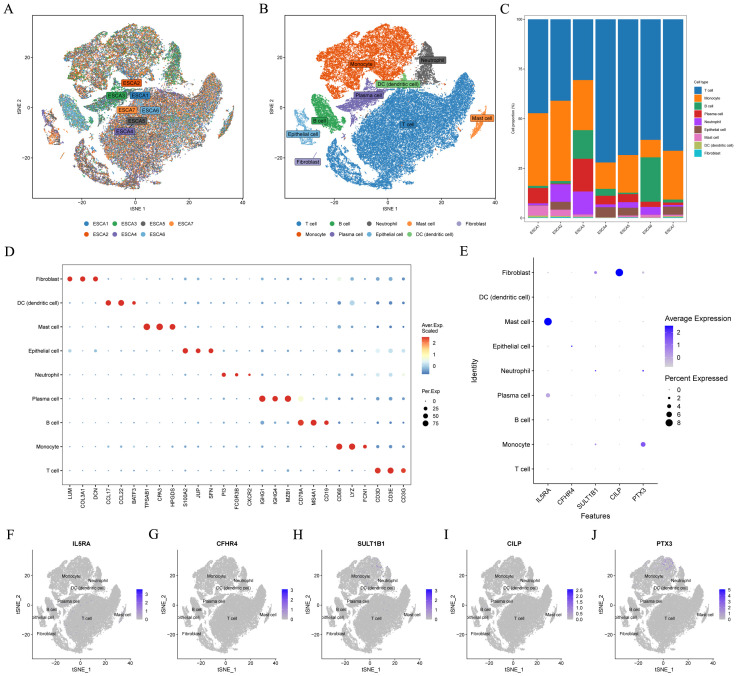
Single-cell sequencing data analysis. In the GSE145370 dataset (n = 7), **(A)** tSNE plot of cell distribution in 7 patients with ESCC. **(B)** tSNE plot for visualizing clustering profiles. **(C)** Proportion of each cell population in different samples. **(D)** Heatmap showing the top 3 unique marker genes in each cellular subpopulation. **(E–J)** The five model CRGs levels in each cellular subpopulation. ESCC, esophageal squamous cell carcinoma; t-SNE, t-distributed stochastic neighbor embedding.

**Figure 10A** depicted the integration results of 7 ESCC patients after eliminating the batch effect. Subsequently, the cells were classified into nine clusters (**Figure 10B**), and **Figure 10D** illustrated the three marker genes in each cell clusters. Moreover, the histogram presented the proportions of cell clusters in the 7 samples (**Figure 10C**). Finally, the expression patterns of five model CRGs were analyzed (**Figures 10E–J**), and FAM151A was not detected in this dataset. The research findings showed that the expressions of five model CRGs exhibited significant differences. Specifically, SULT1B1 was predominantly expressed in fibroblasts.

### 3.9 SULT1B1 inhibits ESCC tumor proliferation and migration

**Figure 11:**
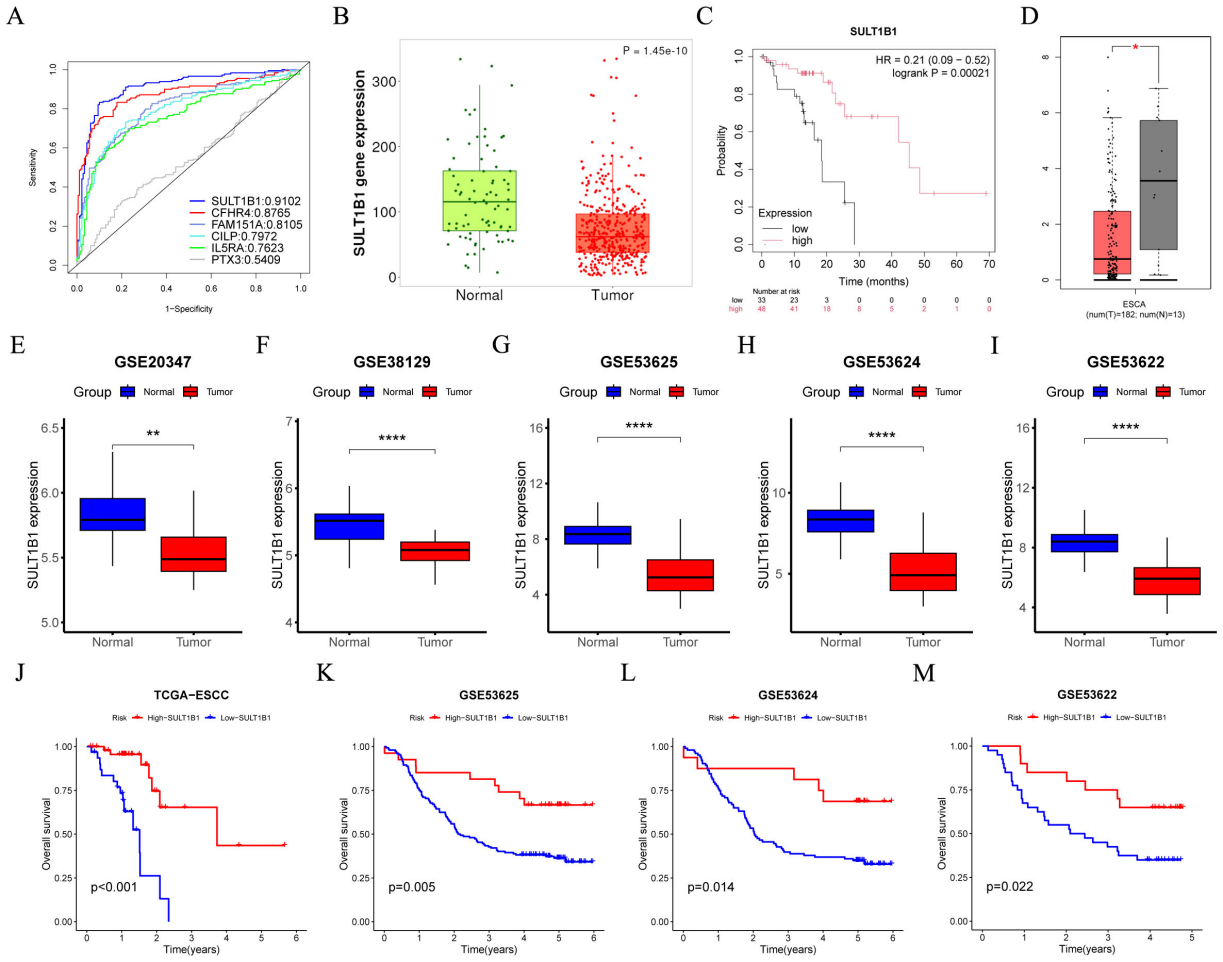
Low expression of SULT1B1 is associated with poor prognosis in ESCC. **(A)** In the GSE53625 cohort (tumor samples = 179 and normal samples = 179), the six model CRGs were analyzed with the ‘pROC’ R package. **(B, D)** The mRNA expression level of SULT1B1 in esophageal cancerous tissues and normal tissues were assessed using the GEPIA and TNMplot databases. **(C)** The Kaplan-Meier survival curve depicted different overall survival (*p* = 0.00021) between the high- and low-SULT1B1 groups using Kaplan-Meier plotter database. **(E–I)** Boxplots of the difference in the mRNA expression level of SULT1B1 between tumor and normal groups across the GSE20347, GSE38129, GSE53625, GSE53624, and GSE53622 cohorts. **(J–M)** The Kaplan-Meier survival curve depicted different overall survival between the high- and low-SULT1B1 groups across the TCGA-ESCC, GSE53625, GSE53624, and GSE53622 cohorts. ESCC, esophageal squamous cell carcinoma. *: *p* < 0.05, **: *p* < 0.01, ****: *p* < 0.0001.

**Figure 12:**
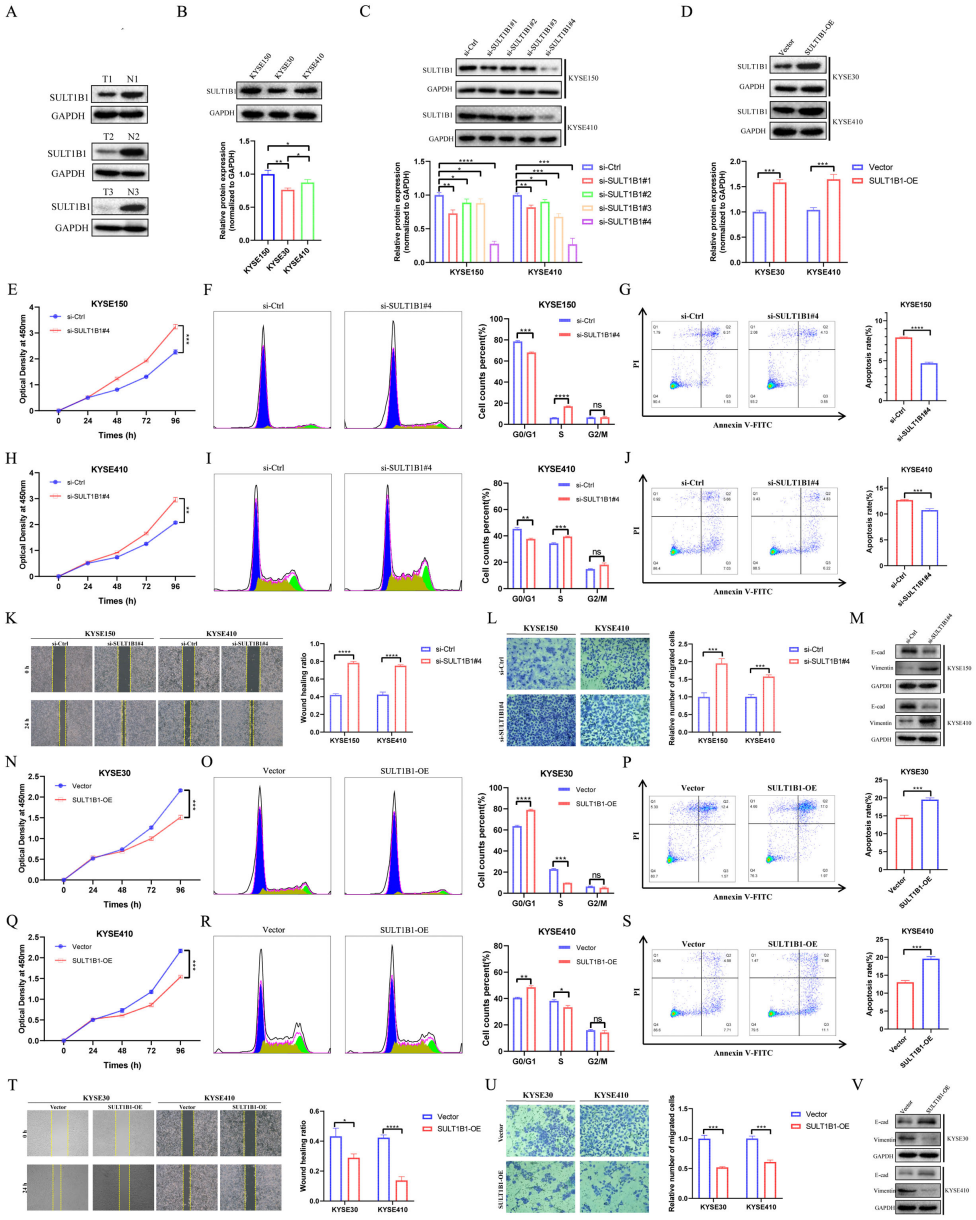
Effects of SULT1B1 on cell proliferation and migration in ESCC cell lines. **(A)** The expression of SULT1B1 protein in ESCC tissues and pericarcinomatous tissues was detected via western blot. **(B)** The protein expression levels of SULT1B1 in various ESCC cell lines with statistical analysis. **(C)** Western blot experiment validated the siRNA knockdown effect in KYSE150 and KYSE410 cells with statistical analysis. **(D)** Western blot experiment validated the SULT1B1 overexpression in KYSE30 and KYSE410 cells with statistical analysis. **(E, H, N, Q)** The results of CCK-8 assay in ESCC cells. **(F, I, O, R)** The effect of knockdown and overexpression of SULT1B1 on the cell cycle of ESCC was detected by flow cytometry. **(G, J, P, S)** The effect of knockdown and overexpression of SULT1B1 on the apoptosis of ESCC was detected by flow cytometry. **(K)** The results of scratch wound healing assay of KYSE150 and KYSE410 cells treated with siRNA or negative control of SULT1B1. **(L)** The results of transwell assay carried out in KYSE150 and KYSE410 cells treated with siRNA or negative control of SULT1B1. **(M)** Expression of E-cad and Vimentin in si-Ctrl group and si-SULT1B1 group in KYSE150 and KYSE410 cells via western blot. **(T)** The results of scratch wound healing assay of KYSE30 and KYSE410 cells with SULT1B1 overexpression. **(U)** The results of transwell assay carried out in KYSE30 and KYSE410 cells with SULT1B1 overexpression. **(V)** Expression of E-cad and Vimentin in vector group and SULT1B1-OE group in KYSE30 and KYSE410 cells via western blot. ESCC, esophageal squamous cell carcinoma. E-cad, E-cadherin. *: *p* < 0.05, **: *p* < 0.01, ***: *p* < 0.001, ****: *p* < 0.0001.

In the GSE53625 cohort, consisting of 179 tumor samples and 179 normal adjacent samples, the capacity of six model CRGs to predict tumor states was assessed utilizing ‘pROC’ R package. The ROC results indicated that among the six model CRGs, SULT1B1 exhibited the highest accuracy in predicting the tumor status (**Figure 11A**). Consequently, we selected it as the focal point and investigated its role in ESCC. Initially, by accessing various online databases, such as GEPIA (http://gepia.cancer-pku.cn/), TNMplot (https://tnmplot.com), and Kaplan-Meier plotter (http://kmplot.com), we analyzed the expression level and prognosis of SULT1B1 in ESCC patients. The analysis revealed that, in comparison with normal tissues, a notable down-regulation of SULT1B1 was detected in ESCC cancer tissues (**Figures 11B, D**). Additionally, when compared with the SULT1B1-lower group, higher SULT1B1 patients expression presented better OS (**Figure 11C**). Furthermore, we conducted a comparison of the expression of SULT1B1 across five transcriptome cohorts retrieved from the GEO databases. The findings indicated that, in comparison with normal tissues, SULT1B1 was significantly down-regulated in ESCC cancer tissues (**Figures 11E–I**). Moreover, in the four prognostic cohorts, it was noted that, in compared to the SULT1B1-lower group, ESCC patients with higher SULT1B1 expression demonstrated better OS (**Figures 11J–M**). Subsequently, we examined three pairs of clinical ESCC tissue samples. The results showed that compared to the paired normal tissues, ESCC cancer tissues demonstrated decreased SULT1B1 expression (**Figure 12A**). The collected data proposed that SULT1B1 might have a tumor-suppressing function in the progression of ESCC.

To investigate the role of SULT1B1, we initially assessed the basal protein expression levels of SULT1B1 in KYSE150, KYSE30, and KYSE410 (**Figure 12B**). The results indicated that SULT1B1 exhibited high expression in KYSE150 cell line, moderate expression in KYSE410 cell line, and low expression in the KYSE30 cell line. Subsequently, SULT1B1 was knocked down in the KYSE150 and KYSE410 cell lines (**Figure 12C**), while it was overexpressed in the KYSE30 and KYSE410 cell lines (**Figure 12D**). The efficiency of overexpression and knockdown was substantiated by western Blot analysis. Here, the subsequent experiments utilized si-SULT1B1#4, as it exhibited a higher level of knockdown efficacy. The CCK8 assay results demonstrated that the knockdown of SULT1B1 was capable of promoting the tumor cell proliferation of KYSE150 and KYSE410 cells (**Figures 12E, H**). Conversely, the overexpression of SULT1B1 was able to suppress the proliferation of KYSE30 and KYSE410 cells (**Figures 12N, Q**). Flow cytometry analysis revealed that upon knockdown of SULT1B1 in KYSE150 and KYSE410 cells, the proportion of cells in the G0/G1 phase showed a decline, whereas the proportion of cells in the S phase exhibited an increase (**Figures 12F, I**). Additionally, the apoptosis rate decreased (**Figures 12G, J**). Conversely, after the overexpression of SULT1B1 in KYSE30 and KYSE410 cells, the proportion of cells in the G0/G1 phase showed an upward trend, whereas the proportion of cells in the S phase declined (**Figures 12O, R**). Moreover, the apoptosis rate increased (**Figures 12P, S**). Results from the scratch assay and transwell invasion experiments confirmed that knockdown of SULT1B1 promoted the migration of KYSE150 and KYSE410 cells (**Figures 12K, L**), while overexpression of SULT1B1 inhibited the migration of KYSE30 and KYSE410 cells (**Figures 12T, U**). The findings of western Blot analysis indicated that upon the knockdown of SULT1B1, the expression of E-cadherin significantly decreased, while that of Vimentin significantly increased (**Figure 12M**). Conversely, when SULT1B1 was overexpressed, an opposite outcome was observed (**Figure 12V**). In summary, the findings of our research indicated that SULT1B1 is capable of effectively suppressing the proliferation and migration of ESCC cells. Further mechanistic investigations confirmed that its tumor-suppressing function is achieved through promoting cell cycle arrest at the G0/G1 phase, inducing apoptosis, and suppressing the epithelial-mesenchymal transition (EMT) process.

## 4 Discussion

ESCC is a kind of malignant tumor characterized by a high incidence and mortality rate. Given the complexity and high heterogeneity of ESCC, solely relying on the clinical and histopathological characteristics of patients does not adequately predict the prognosis of ESCC patients. Due to the remarkable progress of bioinformatics technology, it has now become practicable to predict the prognosis of patients via genetic analysis (34, 35). Hypercoagulable state is highly prevalent in malignant tumors (16, 17). This state facilitates the proliferation and migration of tumor cells, along with the remodeling of the immune microenvironment (18–21). Recent studies indicate that the CRGs signature is highly significant in forecasting the outcomes for patients with diverse cancers, such as colon adenocarcinoma (15), hepatocellular carcinoma (21), and lung adenocarcinoma (16), as well as the responses to immunotherapy. Nevertheless, the role it plays ESCC remains elusive. In this study, we carried out comprehensive analyses and validations across multiple ESCC cohorts, and successfully developed a novel CRGs prognostic signature. When compared with the previously reported 32 prognostic signatures, the CRGs signature demonstrated superior performance over most of the other published signature in ESCC prognostic cohorts. In summary, this signature has been utilized across multiple cohorts, and its effectiveness as a prognostic marker and for examining the efficacy of immunotherapy responses has been preliminarily verified, with the ultimate goal of enhancing the OS of ESCC patients.

In this research, we initially identified prognostic CRGs in patients with ESCC. Next, patients were sorted into two different clusters with cluster 1 having a poorer prognosis. By analyzing the DEGs between different clusters, we constructed a CRGs signature composed of six genes: PTX3, CILP, CFHR4, SULT1B1, IL5RA, and FAM151A. The efficacy of CRGs signature as prognostic predictors was validated in the training and multiple validation cohorts. Among the six model CRGs, SULT1B1 demonstrates the highest accuracy in predicting the tumor status. The SULT family of enzymes is involved in catalyzing the sulfonation process for a wide range of internal, medicinal, and foreign compounds (36). This family encompasses three subfamilies, namely SULT1, SULT2, and SULT4, encompassing a total of 13 distinct members (37). SULT1B1 is a member of the SULT1 family. Research has revealed that the expression level of SULT1B1 is highest in the human intestine. Moreover, it is moderately expressed in the human liver, kidney, lung, and white blood cells (38–40). Despite the role of SULT1B1 in ESCC remains uninvestigated, it is postulated to be associated with carcinogenesis (41). Moreover, numerous studies conducted in recent years have revealed that SULT1B1 might exhibit tumor suppressive activity in a diverse range of cancers. For instance, Eskandarion, M. R. et al. (42) discovered that SULT1B1 exhibited downregulation in gastric cancer and upregulation in intestinal metaplasia. This finding implies that SULT1B1 may possess tumor-suppressive activity during GC progression. In Cholangiocarcinoma, SULT1B1 is closely associated with tumor differentiation. Notably, in CCA, SULT1B1 is lowly expressed (43). Regarding colorectal cancer, the suppression of SULT1B1 is closely linked to tissue dedifferentiation. Moreover, the low expression level of SULT1B1 is associated with a poor survival rate (40, 44–46). In our investigation, through online databases and multiple cohorts, we discovered that the mRNA level of SULT1B1 in ESCC tissues was decreased, and low SULT1B1 was linked to poor survival rates. Correspondingly, the experimental findings indicated that, in ESCC tissues, the SULT1B1 protein levels were remarkably lower. Additionally, our cell-based experiments demonstrated that SULT1B1 exerts a tumor-suppressing effect by modulating cell proliferation and migration.

Recently, immunotherapy has emerged as an effective and highly promising treatment modality for cancer therapy (47, 48). Notwithstanding, only a limited number of patients derive benefits from it (49). Consequently, accurate prediction is of crucial for identifying those patients who are likely to respond favorably to immunotherapy. Our findings indicated that immunotherapy is more likely to elicit a positive response in patients with low-risk ESCC. Additionally, numerous studies have indicated that TMB levels could increase the potency of IC inhibitors (50–53). In this study, patients with low-risk ESCC exhibited higher TMB levels and demonstrated a more robust response to immunotherapy. This finding is in line with the conclusion mentioned above. Moreover, considering the TME’s significant influence on tumor growth and evolution (54, 55), a comprehensive investigation of the TME associated with signature in ESCC is conducive to elucidating its function in the anti-tumor immune response (10). The results showed that, within high-risk subgroup, the level of immune infiltration was higher, with a significant increase in fibroblasts and M0 macrophages. Notably, Cancer-associated fibroblasts, as the fundamental parts of TME, are vital to the development of cancer (56). Research has repeatedly that Cancer-associated fibroblasts can secrete various matrix metalloproteinases and other proteases to remodel the extracellular matrix, thereby leading to tissue hardening, promoting tumor survival, invasion and metastasis, therapy resistance, and immune exclusion (57–62). Furthermore, notable intergroup disparities in IC expression were observed. Given that IC play a critical importance in determining the efficacy of immunotherapy (10, 63), herein we postulate that this could potentially be one of the contributing factors underlying the differences in immunotherapy responses between risk groups. In summary, multiple cohorts have confirmed that CRGs signature is effective in predicting the response to immunotherapy. It should be noted that there is a lack of relevant data regarding immunotherapy in the ESCC cohorts. The aforementioned conclusion was derived through bioinformatics analysis and lacks comprehensive validation in a real ESCC cohort.

Our research has certain limitations. First, in this study, the immunotherapy response prediction is algorithmic. The effectiveness of the CRGs signature in predicting the response to immunotherapy in real clinical environment remains to be validated. Second, while the predictive significance and effectiveness of the CRGs signature have been validated, given that the sample size of the publicly available dataset remains limited, it is essential in larger real-world ESCC cohorts. Third, owing to the limited experimental conditions, this study was unable to further elucidate the molecular mechanisms of the model genes in ESCC. In subsequent research, additional investigations are required to confirm the CRGs signature and delve into the underlying mechanisms.

In conclusion, this study systematically analyzed CRGs related to ESCC via a series of bioinformatics approaches. Subsequently, a robust CRGs signature consisting of PTX3, CILP, CFHR4, SULT1B1, IL5RA, and FAM151A was successfully constructed and validated. Additionally, the CRGs signature is also closely linked to the clinical features, TME, and immunotherapy response of ESCC, holding potential guiding implications for personalized clinical decision-making. Moreover, we have initially elucidated the tumor-suppressing function of SULT1B1 in ESCC. However, it should be noted that the immunotherapy response prediction is algorithmic. Looking ahead, it is essential to validate the prognostic accuracy and the efficacy of immunotherapy responses of the CRGs signature in larger real-world ESCC cohorts.

## Data Availability

Publicly available datasets were analyzed in this study. This data can be found here: Publicly available datasets were derived from GEO (accession number GSE53625, GSE53644, GSE53622, GSE20347, GSE38129, and GSE145370) and TCGA.
