# Supplementary material for: Multi−cohort validation based on coagulation-related genes for predicting prognosis of esophageal squamous cell carcinoma
Source: Front Immunol. 2025 Nov 26;16:1662599. doi: 10.3389/fimmu.2025.1662599 (PMC12689953; doi:10.3389/fimmu.2025.1662599)
Supplement: Supplementary file 2 [file Table2.docx]

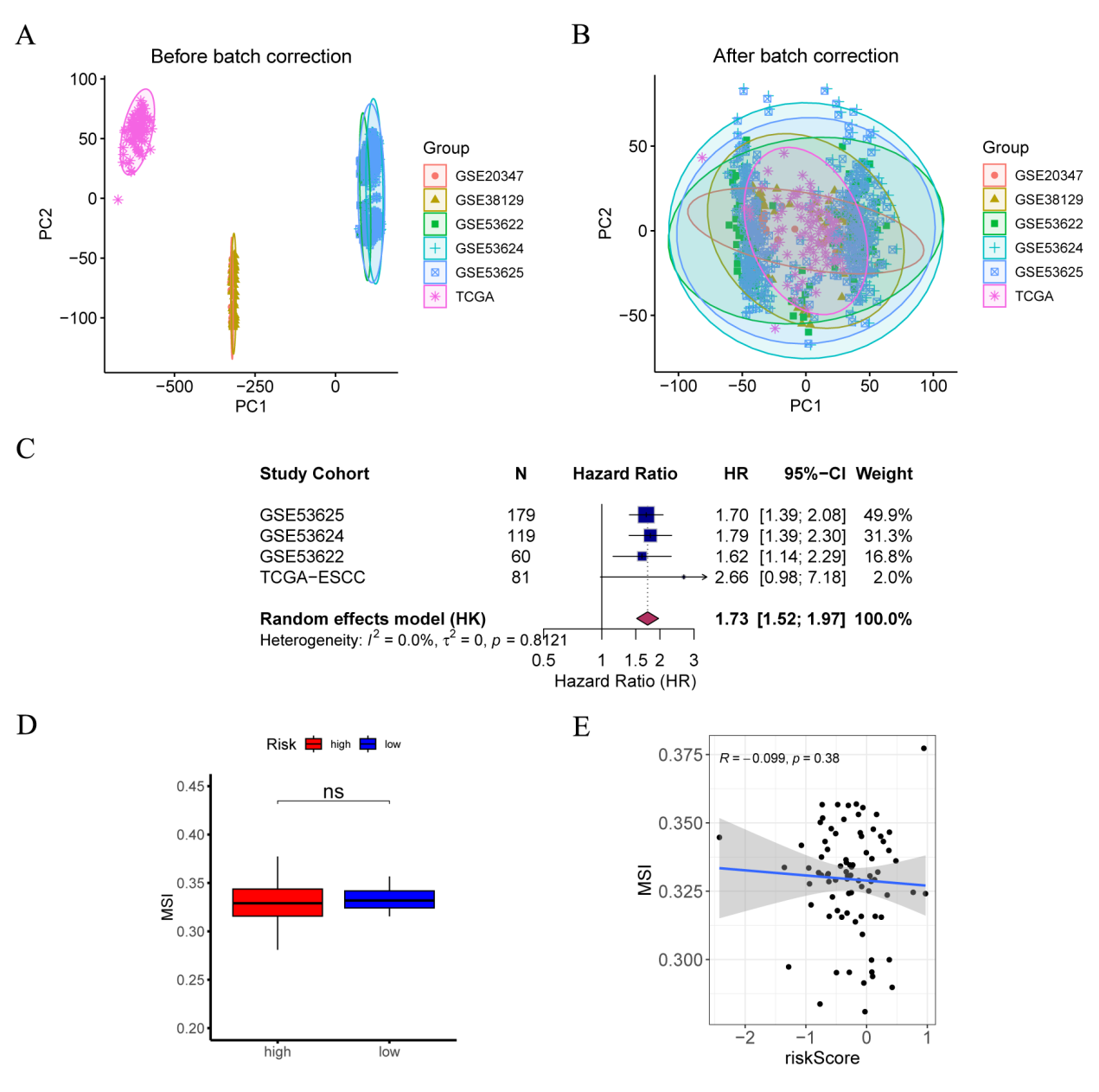


**Supplementary Figure 1.** (A, B) Visualization of removal of batch effects after merging TCGA, GSE53625, GSE53624, GSE53622, GSE20347, and GSE38129 cohorts. (C) A random-effects meta-analysis was conducted on the hazard ratios (HR) across the four cohorts (GSE53625, TCGA-ESCC, GSE53624, and GSE53622). (D) Boxplots of the difference in MSI between the high- and low-risk groups in TCGA-ESCC cohort. (E) The scatter plot showed the correlation between risk score and MSI in TCGA-ESCC cohort. MSI, microsatellite instability; ESCC, esophageal squamous cell carcinoma.
