# Supplementary figures and images for: Multi−cohort validation based on coagulation-related genes for predicting prognosis of esophageal squamous cell carcinoma
Source: Front Immunol. 2025 Nov 26;16:1662599. doi: 10.3389/fimmu.2025.1662599 (PMC12689953; doi:10.3389/fimmu.2025.1662599)

Figure 12A


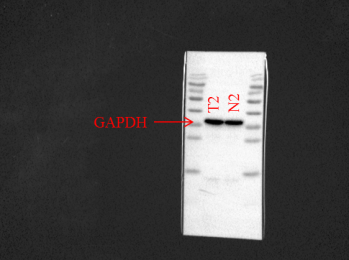

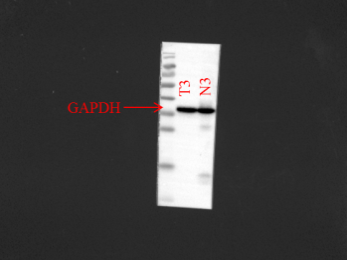

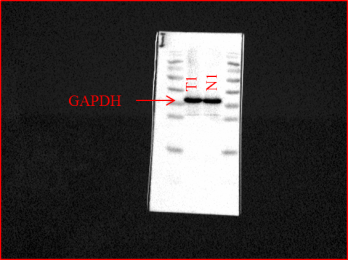


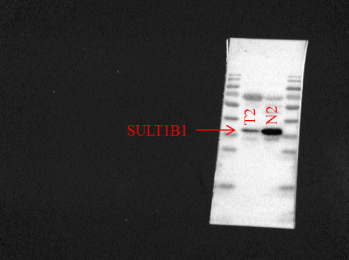

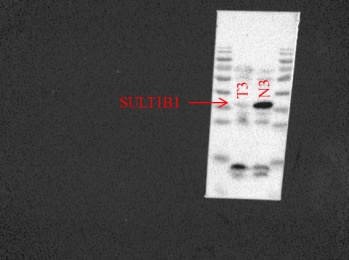

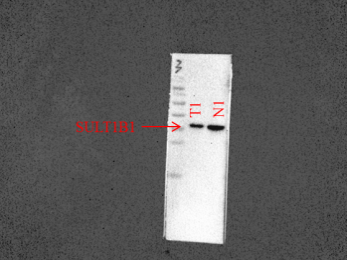


Figure 12B


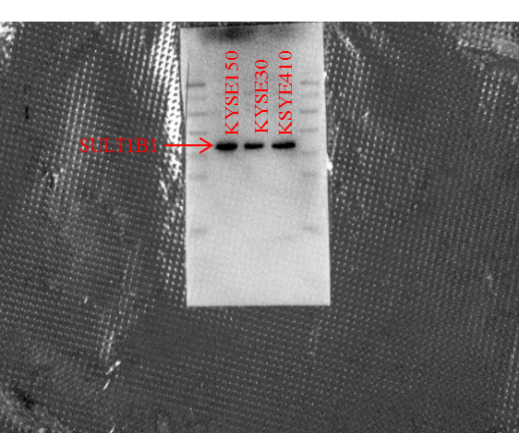

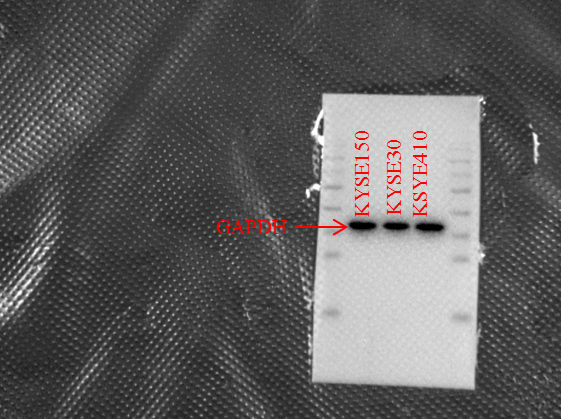


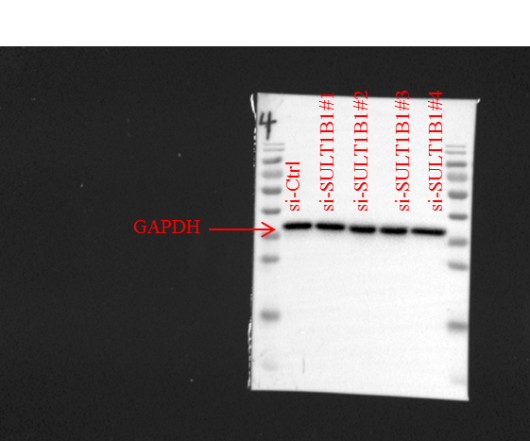

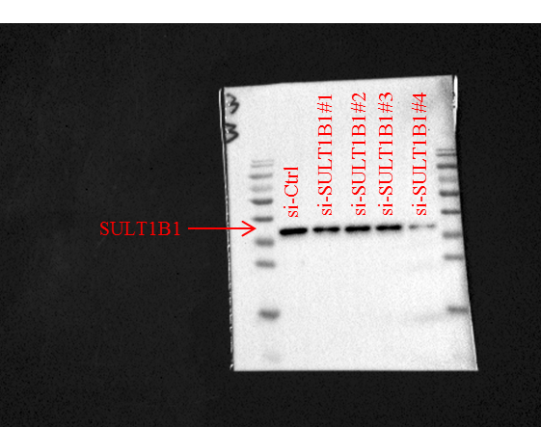
Figure 11C


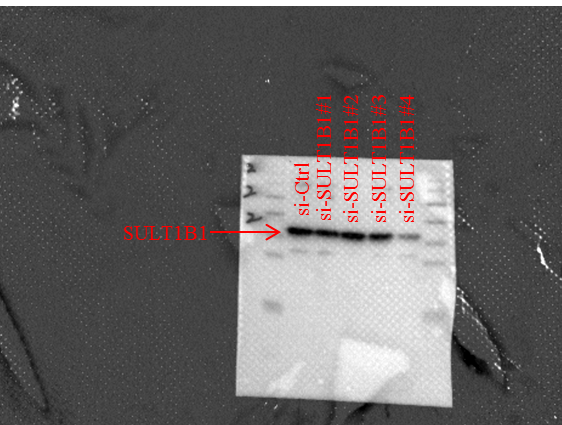

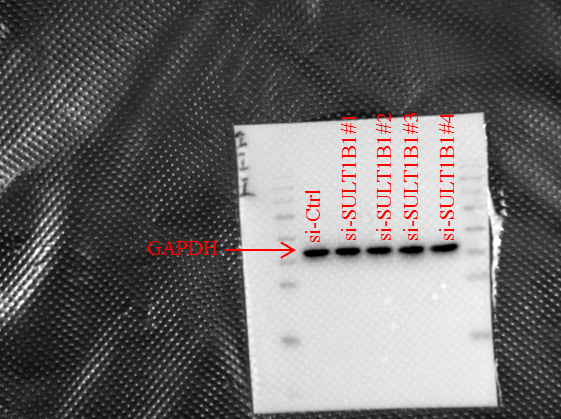


Figure 12D


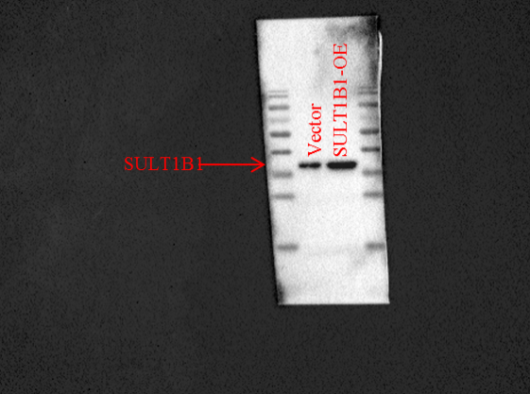

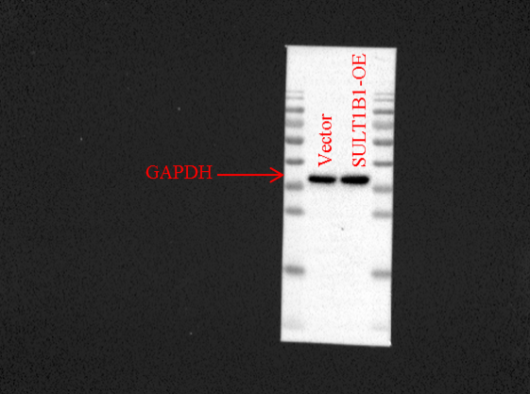


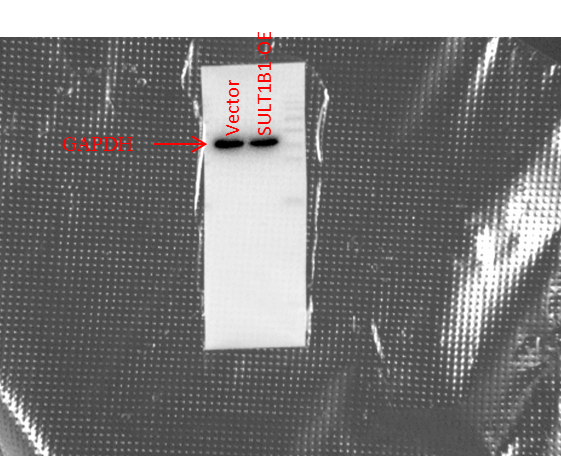

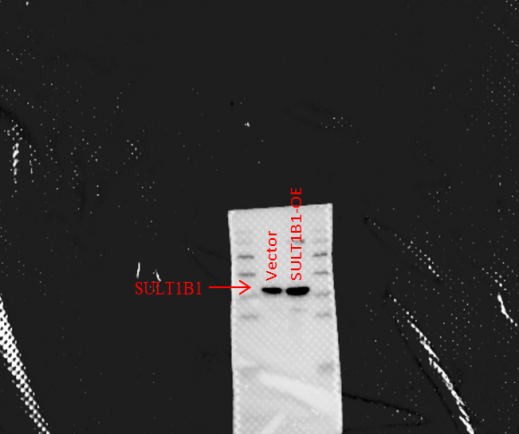


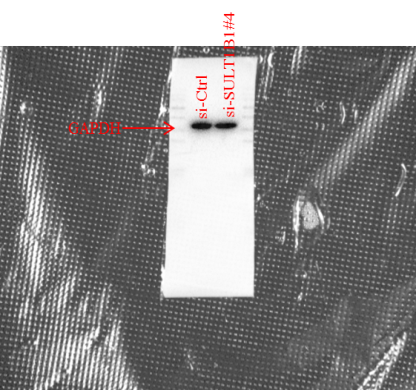

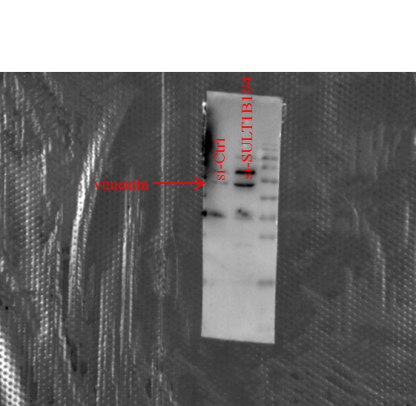
Figure 12M


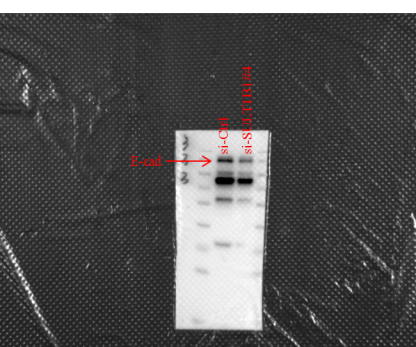


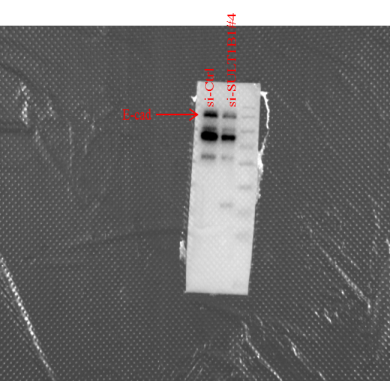


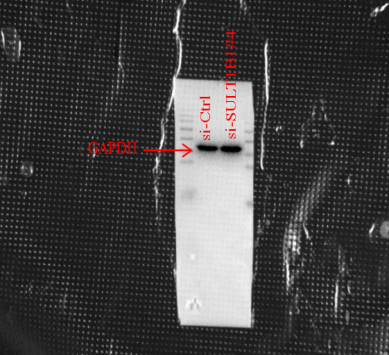

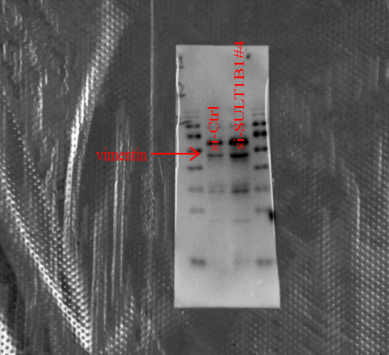


Figure 12V


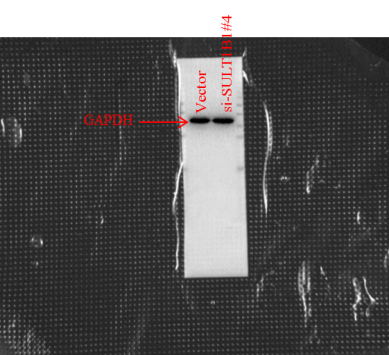

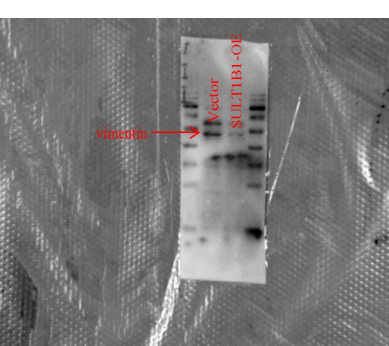

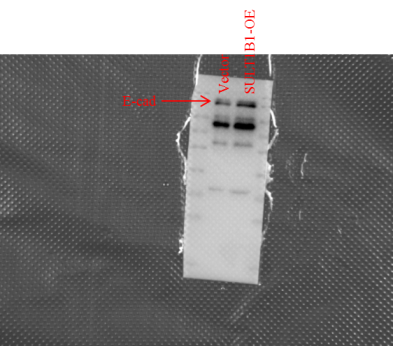


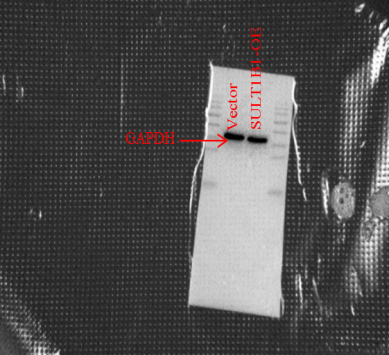

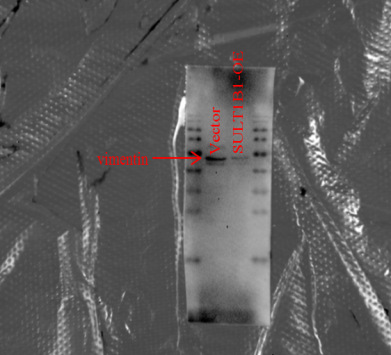

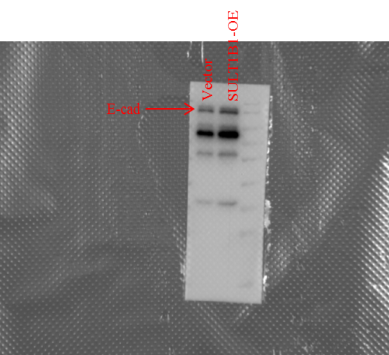

Supplement: Supplementary file 3 [file Table3.docx]
